# Persistent Hyper IgA as a Marker of Immune Deficiency: A Case Report

**DOI:** 10.3390/antib11020030

**Published:** 2022-04-25

**Authors:** Russell J. Hopp, Hana B. Niebur

**Affiliations:** Children’s Hospital and Medical Center, Department of Pediatrics, University of Nebraska Medical Center, Omaha, NE 68198, USA; hniebur@childrensomaha.org

**Keywords:** IgA, immunodeficiency, pediatric

## Abstract

An elevated IgA level obtained in a 10-year-old male a year after an episode of pneumococcal sepsis led to the discovery of a broad-based IgG-specific antibody deficiency syndrome. The specifics of the case and pertinent literature are presented, including a discussion of the hyper-IgD syndrome. An elevated IgA, greater than two standard deviations above the expected age range should prompt a complete workup for selective antibody deficiency syndrome and adds an additional associated marker of an indolent hyper-IgD syndrome in a different clinical circumstance, although the lack of antibody response to vaccines is atypical of the hyper-IgD syndrome.

## 1. Introduction

Hyper-IgA is a rare finding in routine immunoglobulin testing. A single report defines elevated serum IgA at >368 mg/dL for ages 3–16 years based on levels on 12,650 measurements on 6364 pediatric subjects [1]. In adults, an elevated IgA has a different connotation [2,3], even including multiple myeloma [4]. Pediatric IgA levels in excess of 368 mg/dL are often associated with a rheumatological disorder, immune deficiency, or inflammatory gastrointestinal disease, and in most situations, all immunoglobulins are elevated [1]. In fact, the laboratory series reference provided no additional references to support their data [1]. We report here a case of a 10-year-old male with chronic cough and a persistent hyper-IgA following an incidence of pneumococcal bacteremia.

## 2. Case Report

A 10-year-old Hispanic Caucasian male presented to an Allergy-Immunology (A-I) clinic for a second opinion of cough. His symptoms had lasted two years. He had been diagnosed with clinical asthma but the spirometry without albuterol at another office was reported as normal. Allergy tests were negative. His history had included an episode 12 months prior to the A-I visit of pneumococcal bacteremia and his laboratory at hospitalization included a normal IgG and IgM, but an IgA was not performed. His IgE was <2 kU/L. He had received a pneumococcal vaccine after the (in hospital) baseline pneumococcal titers (5/23 > 1.0 micrograms/mL), but a follow-up titer was never obtained. The quantitative lymphocyte subsets: T, B, and NK, and dihydrorhodamine (DHR) flow were drawn at the hospitalization and were normal. He did not have recurrent/periodic fevers.

At the A-I clinic visit, his pre–post albuterol spirometry revealed an 8% improvement in forced vital capacity in 1 s (FEV1) and maxillary sinusitis on plain film. He returned to the clinic 4 weeks later after starting Montelukast and finishing a 3-week course of amoxicillin and clavulanate potassium. His cough was better, but due to the lack of an IgA during his hospitalization, and to perform repeat pneumococcal titers, these tests were obtained. His IgA was 626 mg/dL (a subsequent repeat of 689 micrograms/dL 2 years later; Table 1); his 12-month post-Pneumovax (repeat) pneumococcal titers were unchanged or decreased (3/24 > 1.0 micrograms/mL). His diphtheria titer was non-detectable; his tetanus was 0.1 IU/mL. His *Hemophilus influenza* b titer and varicella titers were all non-detectable, and his rubella titer was protective (Table 1). He had received all his previous routine childhood vaccinations on schedule. He was given repeat pneumococcal, DTap, varicella MMR, and Hib vaccinations, and scheduled for a 4-week follow-up. His IgD level was 23.9 mg/dL (Ref < 15.3).

The repeat immune testing 4 weeks later revealed a non-responsive diphtheria Ab IgG, a rise in tetanus Ab IgG from 0.1 to 0.3 IU/mL, and no rise in VZV Ab IgG; the *H. Influenzae b* Ab IgG fell from 0.1 to negative, and pneumococcal antibodies (23 SEROTYPES) were without a titer change in any of the four previously protective serotypes; the remainder were still low (Table 1). The clinical diagnosis was antibody deficiency with near-normal immunoglobulins or with hyperimmunoglobulinemia (ICD-10 279.05). He was scheduled to receive monthly gammaglobulin. A request for genomic screening for immunoglobulin immunodeficiency or hyper-IgD syndrome was denied by the parents.

## 3. Discussion

Specific elevations of IgA are not common, especially when other immunoglobulins are normal [1,5]. A review of immunodeficiency associated with hypergammaglobulinemia did not mention isolated elevations of IgA [5]. A selective “dysgammagloulinmia” with elevated serum IgA with absent IgG and IgM was reported [6]. In our case, IgG and IgM were normal, but the IgA was markedly increased. Ifmach-Pastore et al. have suggested a hyper-IgA should “raise the suspicion of a serious immune defect, a chronic rheumatological disease or an inflammatory gastrointestinal disorder” [1]. In this regard, a child with a markedly elevated IgA with an inflammatory disorder was reported [7]. Hyper-IgA has been reported in pediatric Henoch Schonlein purpura [8,9] with or without purpura and in periodic fever (hyper-IgD) syndrome [10]. Hyper-IgA has been reported in diabetes [11]. IgA is elevated in the majority of hyper-IgD patients [12]. Our patient did not have other clinical findings of hyper-IgD [12]. Hyper-IgD does not have antibody production deficiencies [12]. The parents refused further (genetic) testing. On his current sub-Q IgG, he has had no further clinical issues.

In support of the finding that hyper-IgA is an unusual co-factor in the antibody deficiency diagnosis is the lack of any vaccination response and the pneumococcal sepsis event. In support of the finding that hyper-IgA is a co-factor in hyper-IgD syndrome is the elevated IgD level [12,13]. The lack of recurrent fevers prior to the pneumococcal sepsis episode (or after) leans away from that diagnosis [12].

Due to having pneumococcal sepsis the previous year, his pneumococcal serotypes had been measured at the initial Allergy-Immunology clinic visit, with only 5 of 23 serotypes showing protection (>1.0 micrograms/mL). His IgM and IgG were normal at the time of hospitalization, but the IgA level had not been obtained. He was given a 23 valent pneumococcal vaccine in the hospital but was not seen till a year later (in the A-I clinic) for the prolonged respiratory issue (cough). At the A-I visit, his repeat pneumococcal tiers were unchanged or lower, compared with the baseline; other vaccination titers were absent or low.

A month following a complete revaccination series, the subject returned for repeat testing. The specific antibody immune hyporesponsiveness persisted (Table 1). He was scheduled for replacement gammaglobulin.

The explanation for the elevated IgA is unknown, but with the defective pneumococcal antibodies (and other antibodies), evidence pointed to an accelerated class switch to IgA. The fact the IgA stayed high over a two-year period suggests a permanent elevation and not a consequence of a vaccination response. One report of patients with hyper-IgA showed increased plasma cells secreting IgA [13]. Whether the elevated IgA improves bacterial protection is speculative. Since IgD is an early phase immunoglobulin [14], and IgA is the result of a terminal class switch process [15], the presence of these two elevated immunoglobulins, along with the deficiency of antibody production (IgG) in our patient, argues this is not a hyper-IgD variant.

## 4. Conclusions

In summary, a markedly elevated IgA was the indication for a subsequently discovered immune dysfunction in a pre-adolescent boy with a previous pneumococcal sepsis episode. The presence of an elevated IgD suggested hyper-IgD syndrome, but the lack of antibody responses to vaccines has not been reported in that syndrome [12]. The parents declined genetic testing. This unique situation of hyper-IgA/IgD and antibody deficiency after repeat vaccinations had not been previously reported in the literature.

## Figures and Tables

**Table 1:** Vaccinations and antibody response.

	Date		Date	Date	Date	Date
Test	1 July 2018	27 July 2018	5 October 2019	19 October 21	19 November 2021	10 August 2020
IgA (mg/dL)	ND	Pneumococcal vaccine	626	Pneumococcal vaccine	657	689
IgG (mg/dL)	609			Dtap		
IgM (mg/dL)	87			MMR		
Positive titer pneumococcal serotypes (23) > 1.0 micrograms/mL	5		3	*H. Flu B*	2	
Negative titer pneumococcal serotypes (23)	18		20	Varicella	21	
Tetanus titer IU/mL	ND		0.1		0.3	
Diphtheria titer IU/mL	ND		0		0	
Rubella titer IU/mL	ND		224		ND	
Hemophilus influenza B titer micrograms/mL	ND		0.1		0	
VZV Ab IgG V	ND		<10		<10	

## Data Availability

Not applicable.
